# *GhWRKY15*, a member of the WRKY transcription factor family identified from cotton (*Gossypium hirsutum* L.), is involved in disease resistance and plant development

**DOI:** 10.1186/1471-2229-12-144

**Published:** 2012-08-12

**Authors:** Feifei Yu, Yifeng Huaxia, Wenjing Lu, Changai Wu, Xuecheng Cao, Xingqi Guo

**Affiliations:** 1 State Key Laboratory of Crop Biology, College of Life Sciences, Shandong Agricultural University, Taian, Shandong 271018, P. R. China; 2 College of Information Science and Engineering, Shandong Agricultural University, Taian, Shandong 271018, P. R. China

**Keywords:** *GhWRKY15*, Cotton, Disease resistance, SA, ROS, Plant development

## Abstract

**Background:**

As a large family of regulatory proteins, WRKY transcription factors play essential roles in the processes of adaptation to diverse environmental stresses and plant growth and development. Although several studies have investigated the role of WRKY transcription factors during these processes, the mechanisms underlying the function of WRKY members need to be further explored, and research focusing on the WRKY family in cotton crops is extremely limited.

**Results:**

In the present study, a gene encoding a putative WRKY family member, *GhWRKY15*, was isolated from cotton. *GhWRKY15* is present as a single copy gene, and a transient expression analysis indicated that GhWRKY15 was localised to the nucleus. Additionally, a group of *cis*-acting elements associated with the response to environmental stress and plant growth and development were detected in the promoter. Consistently, northern blot analysis showed that *GhWRKY15* expression was significantly induced in cotton seedlings following fungal infection or treatment with salicylic acid, methyl jasmonate or methyl viologen. Furthermore, *GhWRKY15*-overexpressing tobacco exhibited more resistance to viral and fungal infections compared with wild-type tobacco. The *GhWRKY15*-overexpressing tobacco also exhibited increased RNA expression of several pathogen-related genes, *NONEXPRESSOR OF PR1*, and two genes that encode enzymes involved in ET biosynthesis. Importantly, increased activity of the antioxidant enzymes POD and APX during infection and enhanced expression of *NtAPX1* and *NtGPX* in transgenic tobacco following methyl viologen treatment were observed. Moreover, *GhWRKY15* transcription was greater in the roots and stems compared with the expression in the cotyledon of cotton, and the stems of transgenic plants displayed faster elongation at the earlier shooting stages compared with wide type tobacco. Additionally, exposure to abiotic stresses, including cold, wounding and drought, resulted in the accumulation of *GhWRKY15* transcripts.

**Conclusion:**

Overall, our data suggest that overexpression of *GhWRKY15* may contribute to the alteration of defence resistance to both viral and fungal infections, probably through regulating the ROS system via multiple signalling pathways in tobacco. It is intriguing that *GhWRKY15* overexpression in tobacco affects plant growth and development, especially stem elongation. This finding suggests that the role of the WRKY proteins in disease resistance may be closely related to their function in regulating plant growth and development.

## Background

Although sessile plants lack a circulating, somatically adaptive immune system, they have developed specific mechanisms to respond to diverse environmental signals that may cause stress and restrict growth and development. Among these mechanisms, many genes that either directly protect plants against stress or further control the expression of other target genes are induced or repressed [1]. Therefore, plant defence responses are regulated by a network of signalling pathways that fine-tune the transcriptional activation of defence-related genes. Among these complex responses, it has been demonstrated that the transcriptional activation of defence-related genes requires the expression of particular transcription factors and/or alterations in their DNA-binding activity [2,3].

The WRKY family is one of the largest families of transcription factors in plants. Since the first member was isolated in sweet potato, an increasing number of WRKY members have been identified, including 74 members in *Arabidopsis thaliana* and 109 members in *Oryza sativa*[4,5]. The defining characteristic of WRKY transcription factors is their DNA-binding domain, known as the WRKY domain, which is composed of a highly conserved peptide sequence (WRKYGQK) and a zinc-finger motif, (either Cx_4-5_Cx_22-23_HxH or Cx_7_Cx_23_HxC) [6]. Moreover, the conservation of the WRKY domain is mirrored by the conservation of cognate *cis*-acting W-box elements ((T)TGAC(C/T)) in the target promoter region, which are essential for the function and binding of the WRKY protein [4]. The number of highly conserved WRKY domains and the structure of the zinc-finger motif have resulted in the division of the WRKY superfamily into three distinct groups (I, II and III), and group II can be further split into five distinct subgroups (IIa to IIe) based on the presence of additional short conserved structural motifs [4].

Previous studies have examined the roles of plant WRKY proteins in response to pathogens, and WRKY proteins can function as either positive or negative regulators of the defence response. Constitutive overexpression of *WRKY70* results in increased resistance to pathogens and the constitutive expression of salicylic acid (SA)-induced pathogen-related (PR) genes. Generally, the expression of *AtWRKY70* is activated by SA and repressed by JA [7]. Furthermore, the enhanced resistance of *CaWRKY1*-silenced chilli pepper leaves to *Xanthomonas axonopodis* pv*. vesicatoria* suggests that *CaWRKY1* acts as a negative regulator of the defence response [8]. Interestingly, a dual function in defence signalling has been suggested for *AtWRKY53* and *AtWRKY41*[9,10]. *WRKY41*-overexpressing *Arabidopsis* exhibited enhanced resistance to *Pseudomonas syringae* but increased susceptibility to *Erwinia carotovora*[10]. Mechanistically, to increase the defence response, WRKY transcription factors frequently upregulate the expression of several pathogen-related genes and *NONEXPRESSOR OF PR1* (*NPR1*) by binding specifically to the W-box element in their promoter region [3,11]. The expression of PR proteins, including antimicrobial chitinases and glucanases that directly destroy the cell walls of fungal pathogens, is temporarily and spatially activated following pathogen recognition [12]. NPR1 is a key regulator of the SA-dependent defence response and systemic acquired resistance (SAR), and plants under-expressing *NPR* gene are more susceptible to infection with pathogens [3]. These data suggest that the mechanism underlying the regulation of the defence response by WRKY transcription factors is fairly complex and needs to be examined further.

Importantly, GmWRKY27 proteins in soybean (*Glycine max* L.) probably form homodimers and can efficiently activate reporter gene expression in a two-hybrid assay [13]. Interactions among WRKY18, WRKY40 and WRKY60 can result in both homocomplexes and heterocomplexes, thereby allowing for adaptation to different microbial pathogens [14]. Such protein-protein interactions may affect properties that are important for the regulatory functions of these proteins [14].

Few studies have examined the role of the WRKY family of transcription factors in plant development. The overexpression of *GmWRKY13* promotes lateral root development, and this promotion is probably the result of the activation of the downstream gene *ARF6*, a component of the auxin signalling pathway [13]. Some WRKY transcription factors also function in seed development, dormancy and germination; leaf senescence; and trichome development, which are mostly dependent upon the abscisic acid (ABA) and gibberellin (GA) signalling pathways [15-18]. A recent study has demonstrated that the *wrky12 A. thaliana* mutants underwent secondary thickening in the walls of certain pith cells that were associated with the ectopic deposition of lignin, xylan and cellulose, leading to an approximately 50% increase in biomass density in stem tissues. This finding suggests the possibility of significantly increasing the mass of fermentable cell wall components in bioenergy crops [19].

Together, the above findings suggest that the WRKY transcription factors play a crucial role in plant development and disease resistance [6]. Whereas previous research has been largely restricted to *Arabidopsis*, tobacco and rice, data regarding the WRKY family of transcription factors in cotton (*Gossypium hirsutum* L.), an important economic crop, are notably limited. In the present study, a cDNA clone, *GhWRKY15*, encoding a putative IId WRKY gene, was isolated and characterised, and the gene expression patterns under various biotic and abiotic stresses were investigated. We obtained information on the potential of overexpression of *GhWRKY15* to alter responses to fungal or viral pathogens and developmental processes in tobacco. This study suggests the alteration of responses to disease attacks in *GhWRKY15* overexpressing tobacco may be related to multiple defence signalling pathways, and further via regulation of the reactive oxygen species (ROS) network.

## Results

### Cloning and characterisation of *GhWRKY15*

Due to the vital function of the WRKY motif in the activity of the WRKY family members, we designed a pair of degenerate primers targeting this region (DP1 and DP2, see Additional file 1: Table S2) in an attempt to isolate WRKY family members from cotton. Using this primer pair, the internal conserved region of one IId WRKY subfamily member was obtained. Next, rapid amplification of cDNA ends PCR (RACE-PCR) was used to identify the full-length cDNA, and a 419-bp 5' untranslated region (UTR) and a 546-bp 3' UTR were amplified. To verify the full-length cDNA sequence, two specific primers were designed, and sequence analysis revealed that the full-length cDNA contained a 942-bp open reading frame (ORF) encoding a 314-amino acid protein with a predicted molecular mass of 34.872 kDa and an isoelectric point of 10.07. Because this putative cotton WRKY family member shares a high degree of homology with WRKY15 in *A. thaliana*, we designated this gene *GhWRKY15* (GenBank: GU207867).

**Figure 1:**
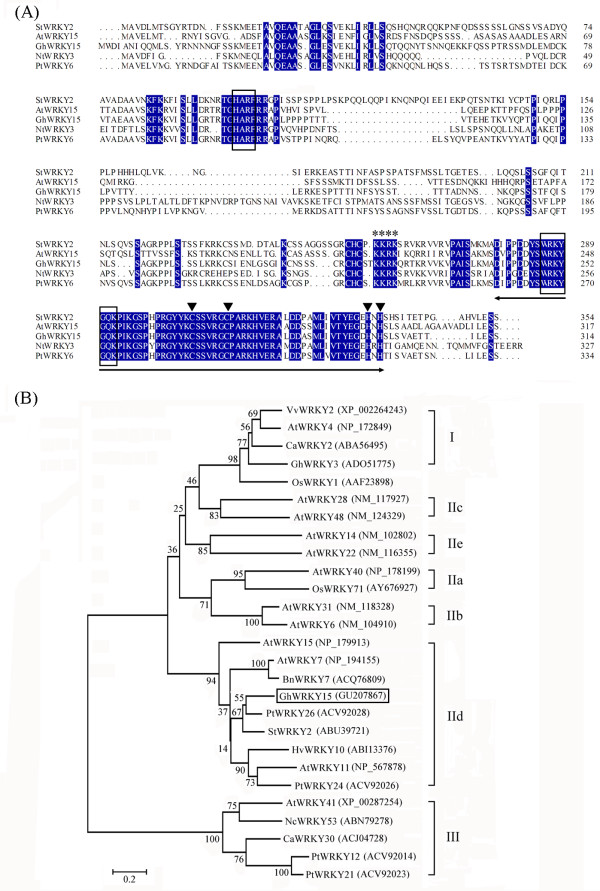
**Characterisation of WRKY transcription factors from various species.** (**A**) Identical amino acids are highlighted in blue. The approximately 60-amino acid WRKY domain and the C and H residues in the zinc-finger motif (C-X_4-5_-C-X_22-23_-H-X_1_-H) are marked by the *two-headed arrow* and *triangle*, respectively. The short conserved HARF structural motif and the highly conserved amino acid sequence WRKYGQK in the WRKY domain are boxed. (**B**) Phylogenetic analysis of GhWRKY15 in relation to other plant WRKY transcription factors. The WRKY transcription factors used are as follows: GhWRKY15 (GU207867) and GhWRKY3 (ADO51775) from *G. hirsutum*, PtWRKY26 (ACV92028), PtWRKY24 (ACV92026), PtWRKY12 (ACV92014) and PtWRKY21 (ACV92023) from *P. tomentosa*, StWRKY2 (ABU49721) from *S. tuberosum*, AtWRKY15 (NP_179913), AtWRKY7 (NP_194155), AtWRKY11 (NP_567878), AtWRKY4 (NP_172849), AtWRKY40 (NP_178199), AtWRKY31 (NM_118328), AtWRKY6 (NM_104910), AtWRKY28 (NM_117927), AtWRKY48 (NM_124329), AtWRKY14 (NM_102802), AtWRKY22 (NM_116355), and AtWRKY41 (XP_00287254) from *A. thaliana*, BnWRKY7 (ACQ76809) from *Brassi canapus*, CaWRKY2 (ABA56495) and CaWRKY30 (ACJ04728) from *Capsicum annuum*, HvWRKY10 (ABI13376) from *Hordeum vulgare*, VvWRKY2 (XP_002264243) from *Vitis vinifera*, OsWRKY71(AY676927) and OsWRKY1 (AAF23898) from *O. sativa* and NcWRKY53(ABN79278) from *Noccaea caerulescens.*

A protein sequence comparison between GhWRKY15 and the other plant WRKY proteins demonstrated that the amino acid sequences of these proteins are highly similar, with a homology of 42.60% to NtWRKY3 (BAA77358), 49.70% to AtWRKY15 (NP_179913), 54.27% to StWRKY2 (ABU49721), and 63.19% to PtWRKY16 (ACV92028) (Figure 1A). The predicted GhWRKY15 protein contains an approximately 60-amino acid WRKY domain that is composed of the conserved amino acid sequence (WRKYGQK) and a zinc-finger motif (C-X_4-5_-C-X_22-23_-H-X_1_-H), and a short conserved structural motif (HARF), indicating that GhWRKY15 belongs to Group IId of the WRKY family [20]. Additionally, a putative nuclear localisation signal (NLS), KKRK, was found at position 220–223 (Figure 1A). 

A phylogenetic analysis was performed to determine the evolutionary relationship between GhWRKY15 and the other WRKY proteins (Figure 1B). Overall, WRKY proteins can be classified into three groups, and Group II can be further divided into five subgroups (IIa, IIb, IIc, IId and IIe). The phylogenetic analysis demonstrated that GhWRKY15 was closely related to Group IId WRKY family members, including PtWRKY26, StWRKY2, BnWRKY7, AtWRKY7 and AtWRKY15. These results strongly suggest that GhWRKY15 belongs to Group IId of the WRKY family, the members of which may share some similar functions in different species.

To examine the genomic organisation of the *GhWRKY15* locus, we amplified the *GhWRKY15* genomic sequence with a pair of specific primers that had been designed based on the full-length cDNA sequence. The full-length *GhWRKY15* genomic sequence was 1306 bp in length, and two short introns (115 bp and 90 bp) that exhibited a high AT content and had a typical 5'-GT splice donor and 3'-AG splice acceptor were detected. The comparative analysis of the *GhWRKY15* (GenBank: GU207869), *AtWRKY7* (NC_003075), *AtWRKY15* (NC_003071) and *VvWRKY* (NW_002239918) genomic sequences revealed that the number and position of the introns were well-conserved (Additional file 2: Figure S1). Specifically, the insert position of the second intron, called an R-type intron, was in the arginine codon of the WRKY domain. Additionally, we also determined the copy number of the *GhWRKY15* locus in cotton using fluorescence real-time quantitative PCR (qPCR) analysis with RNA-dependent RNA polymerase 6 (*GhRDR6*), which is present in the cotton genome as single copy [21], as an internal standard. The standard curve of *GhRDR6* and *GhWRKY15* is shown in Additional file 3 (Figure S2), and the correlation coefficients between the standard curves of *GhRDR6* and *GhWRKY15* were highly significant. As shown in Additional file 1 (Tables S3, S4), our results demonstrate that *GhWRKY15* is probably a single-copy gene in cotton.

### GhWRKY15 is localised to the nucleus

**Figure 2:**
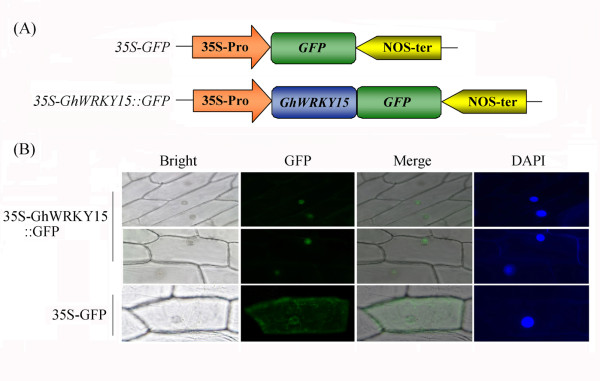
**Subcellular localisation of the GhWRKY15::GFP fusion protein.** (**A**) Schematic representation of the 35 S-GhWRKY15::GFP fusion construct and the 35 S-GFP construct. GFP was fused in frame to the C terminus of GhWRKY15. (**B**) Onion epidermal cells transiently expressing either the 35 S-GhWRKY15::GFP and 35 S-GFP construct were viewed using a confocal laser scanning microscope. The nuclei of the onion cells were visualised using DAPI staining.

To investigate the mechanism underlying the regulatory activity of *GhWRKY15* in development and during the stress response, the subcellular localisation of GhWRKY15 was predicted using the PSORT programme. An NLS (KKRK) was identified in GhWRKY15, suggesting that it is mostly located in the nucleus. To confirm this prediction, a biolistic transformation system was used for a transient assay, and two constructs, *35S-GhWRKY15::GFP* and *35S-GFP* (Figure 2A), were individually introduced into onion epidermal cells. The fluorescence was observed using a confocal laser scanning microscope with DAPI staining to detect the nuclei and interference contrast images to detect the whole-cell structures. As shown in Figure 2B, the fluorescence was predominantly localised to the nucleus in the onion epidermal cells expressing *35S-GhWRKY15::GFP*, whereas the fluorescence was present throughout the cytoplasm and nucleus in the cells transfected with the control *35S-GFP* construct. These results indicate that the GhWRKY15 protein is located in the nucleus, and this localisation may aid in the determination of both the function and the molecular mechanism underlying the function of this protein.

### Analysis of partial putative *cis*-acting elements in the *GhWRKY15* promoter

A 1012-bp fragment of the *GhWRKY15* promoter (GenBank: GU207869) was obtained using Inverse PCR (I-PCR) and nested PCR. An analysis of this region using the PlantCARE databases revealed a group of putative *cis*-acting elements that suggest that GhWRKY15 may play a role in the response to environmental stress. Specifically, we identified pathogen/elicitor-related elements including RAV1AAT, ARE, W-box and TL1ATSAR, abiotic stress responsive element including ACGTATERD1, HSE, LTR and MBS and tissue-specific and development-related elements including a Skn-1 motif, circadian, OSE2ROOTNODULE, POLLEN1LELAT52 and AACACOREOSGLUB1. All of the identified *cis*-elements are listed in Table 1.

**Table 1:** **Putative *cis *-acting elements of the promoter of *GhWRKY15 ***

***Cis*-element**	**Position**	**Sequence**
*Light responsive elements*		
AE-box	−445 (−)	AGAAACTA
ATCC-motif	−575 (+)	CAATCCTC
Box 4	−362 (+), -1013(+)	ATTAAT
G-box	−752 (+)	GACATGTGGT
I-box	−541 (+)	GATATGG
LAMP-element	−722 (+)	CTTTATCA
Sp1	−4 (+), -31 (−)	CC(G/A)CCC
TCT-motif	−444 (−)	TCTTAC
*Tissue-specific and development-related elements*		
Skn-1_motif	−522 (+), -628 (−)	GTCAT
circadian	−350 (+), -763 (−)	CAANNNNATC
OSE2ROOTNODULE	−808 (+)	CTCTT
POLLEN1LELAT52	−82 (+), -163 (+), -306 (+), -345 (+)	AGAAA
AACACOREOSGLUB1	−17 (+)	AACAAAC
*Pathogen/elicitor-related elements*		
RAV1AAT	−197 (+), -256 (+), -320 (+), -973 (+)	CAACA
ARE	−534 (+), -705 (+)	TGGTTT
W-box	−867 (+)	(T)(T)TGAC(C/T)
TL1ATSAR	−855 (+)	TTCTTCTTCAG
*Abiotic stress responsive element*		
ACGTATERD1	−118 (+)	ACGT
HSE	−301 (+),-609 (+)	A(A/G)AAAATT(T/C)(C/G)
LTR	−465 (−), -632 (+)	CCGAAA
MBS	−295 (−),-908 (+)	TAACTG

### *GhWRKY15* expression in cotton following exposure to diverse biotic stresses, SA, methyl jasmonate (MeJA) and ET

**Figure 3:**
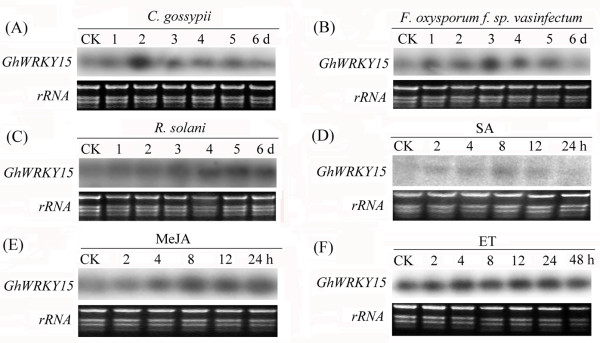
**Expression of *GhWRKY15 *in response to different fungal infections and hormone treatments.** Approximately one-week-old cotton seedlings were used for all treatments. For the fungal inoculation, the roots of the cotton seedlings were dipped into conidial suspensions of *C. gossypii* (**A**), *F. oxysporum* f. sp. *vasinfectum* (**B**) or *R. solani* (**C**) (10^5^ conidia/ml). The signalling molecules used were 2 mM SA (**D**), 100 μM MeJA (**E**) and ET released from 5 mM ethephon (**F**). Whole seedling plants were collected for RNA extraction. Ethidium bromide-stained rRNA was included as a loading control.

To examine the role of *GhWRKY15* in response to biotic stress in cotton, cotton seedlings were infected with three different fungi (*C. gossypii*, *Fusarium oxysporum* f. sp*. vasinfectum* and *Rhizoctonia solani*) using the root dip method. As shown in Figure 3A, B, C and Additional file 4 Figure S3A, B, C, these pathogen treatments dramatically enhanced the accumulation of *GhWRKY15* transcripts. The maximum accumulation of the *GhWRKY15* transcripts occurred 2, 3 and 5 d after treatment with these three fungi, respectively. There was no obvious change in the expression of *GhWRKY15* under normal conditions without any pathogen infection within 7 days. These data suggest that *GhWRKY15* may play important roles in the response to pathogens.

To elucidate the molecular mechanism underlying the responsiveness to various biotic stresses, we examined the expression of *GhWRKY15* following treatment with exogenously applied SA, MeJA and ET, which are all phytohormones involved in different signalling pathways. As shown in Figure 3D, E, F, and Additional file 4 (Figure S3D, E, F), the expression of *GhWRKY15* was obviously enhanced by SA, MeJA (an analogue of JA) and ET. After the SA treatment, *GhWRKY15* expression was noticeably enhanced at 8 h. Following the MeJA treatment, transcripts of *GhWRKY15* accumulated at 4 h and reached a peak from 8 h to 24 h. Under ET treatment, *GhWRKY15* accumulated at 2 h, and reached a peak at 4 h. But ET treatment does not lead to a higher expression than SA or MeJA treatment, respectively. Therefore, *GhWRKY15* may be involved in the SA, ET and JA signalling pathways that function in disease resistance.

### Tobacco plants overexpressing *GhWRKY15* exhibit enhanced viral and fungal resistance

**Figure 4:**
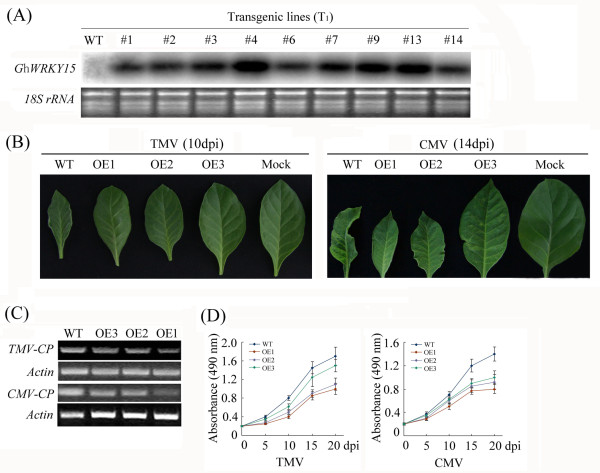
**Enhanced resistance of *GhWRKY15*-overexpressing tobacco to viruses.** (**A**) Northern blot analysis of the expression levels of *GhWRKY15* in T_1_ transgenic and WT tobacco under normal conditions. Two leaves were tested for the *GhWRKY15* transgenic tobacco. WT: wild-type. (**B**) Leaf symptoms of tobacco plants infected with TMV (10 days post-inoculation) or CMV (14 days post-inoculation). OE: *GhWRKY15-*overexpressing tobacco; Mock: mock inoculation; CP: coat proteins. (**C**) RT-PCR analysis of the expression levels of the *CP* gene in infected transgenic lines (OE1, OE2 and OE3) and the WT line. (**D**) TMV and CMV titres in the transgenic lines and the wild-type lines. The data are presented as the mean ± standard error from three independent experiments.

To investigate the effects of constitutive overexpression of *GhWRKY15* on tobacco, full-length *GhWRKY15* was cloned into the binary vector pBI121 by replacing the GUS gene, and tobacco lines overexpressing *GhWRKY15* were generated. A total of 14 independent transgenic lines were obtained following selection with kanamycin, and these lines were confirmed using PCR. Additionally, 10 lines of transgenic T_1_ plants were randomly selected for an expression analysis. The expression of the transformed *GhWRKY15* gene in the leaf tissue was detected using northern blots, whereas the control, wild-type (WT) tobacco lines demonstrated no expression (Figure 4A). Three independent lines, OE1 (6#), OE2 (9#) and OE3 (4#), which exhibited different expression levels of the target gene were used for further functional analysis.

The disease resistance tests were performed on the T_2_ progeny of the three transgenic lines using two kinds of viruses and fungi and compared with the resistance of WT plants. Six-week-old transgenic tobacco plants were inoculated with *tobacco mosaic virus* (TMV) or *cucumber mosaic virus* (CMV). Minor disease symptoms were observed in the WT plants seven days after inoculation with TMV, with the leaf veins and adjacent tissue colour fading and appearing dark green, whereas no symptoms were seen in any of the three transgenic lines. The leaves of the TMV-inoculated lines 10 days after inoculation are shown in Figure 4B. However, there were no significant differences in the disease symptoms between the transgenic and WT plants fourteen days after inoculation with TMV; both plants exhibited severely diseased, crinkled leaves, leaf distortion, plant dwarfism and slow growth. For the CMV inoculation, no difference in the disease symptoms was observed between the WT and transgenic lines up to seven days after the inoculation, but the WT plants exhibited significantly more severe disease symptoms relative to the transgenic plants after fourteen days (Figure 4B). Furthermore, the expression level of the coat proteins (CP) gene was much lower in both the TMV- and CMV-inoculated transgenic plants than in the WT line (Figure 4C). Additionally, as detected using ELISAs, the average expression of the TMV and CMV CPs in the transgenic lines was lower than the expression in the WT line (Figure 4D).

**Figure 5:**
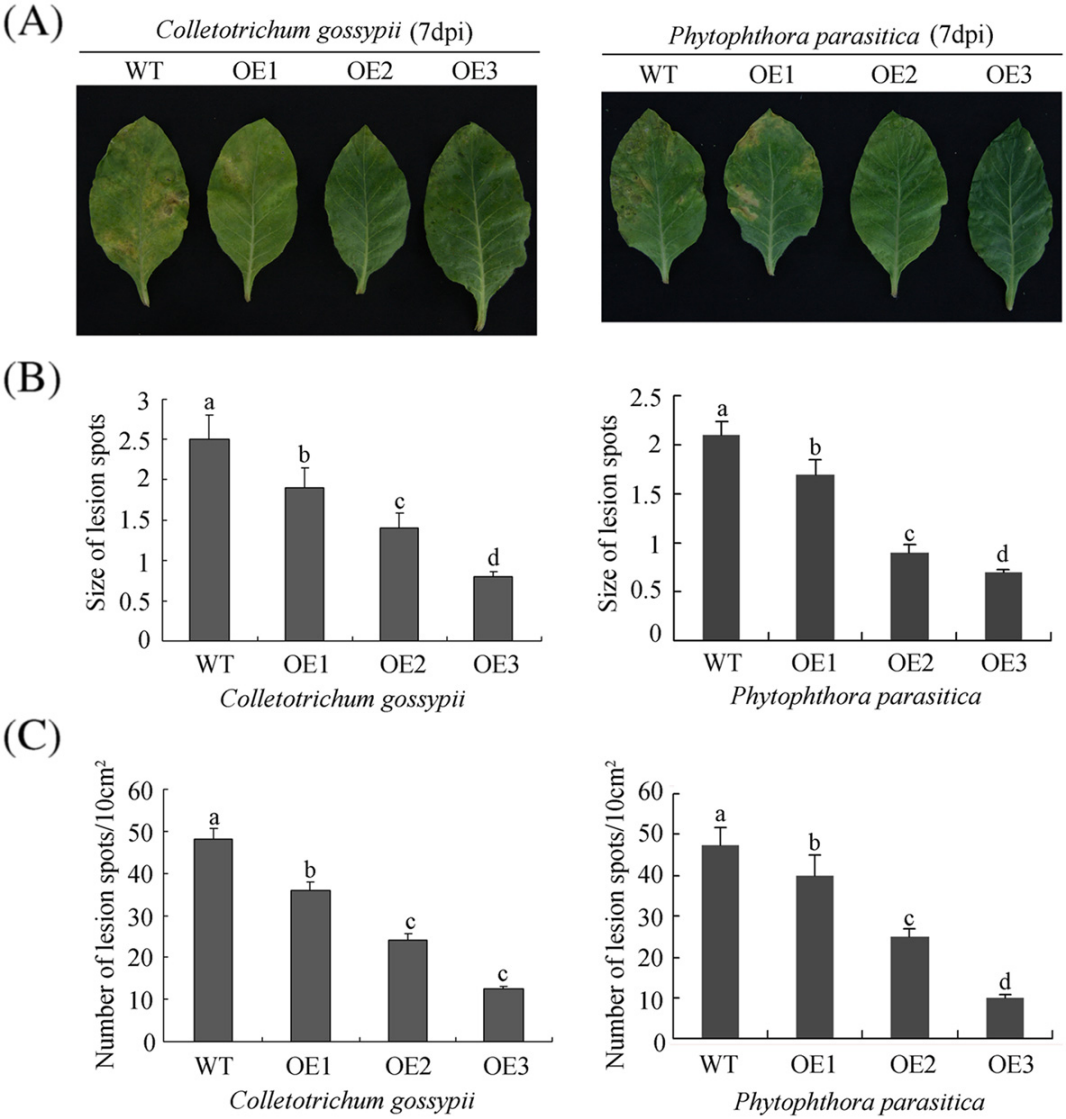
**Enhanced resistance of *GhWRKY15*-overexpressing tobacco to fungi.** (**A**) Leaf symptoms of tobacco plants infected with fungi. The detached leaves in transgenic and wild-type tobacco were inoculated with *C. gossypii* or *P. parasitica* suspensions (10^6^ conidia/ml) prepared in 1% glucose, and the leaves were photographed 7 days after inoculation. (**B**) The diameters of the lesions on the inoculated leaves. The diameters of the lesion spots were recorded using the following scoring system: 0, < 1 mm; 1, 1–2 mm; 2, > 2 mm. (**C**) The numbers of lesions on the inoculated leaves. The number of lesions per 10 cm^2^ was counted on the inoculated leaves of three independent transgenic and wild-type plants. The values indicated by the different letters are significantly different at *P* < 0.05, as determined using Duncan’s multiple range tests.

To investigate whether *GhWRKY15*-overexpressing tobacco plants possess enhanced fungal resistance compared with WT plants, conidial suspensions of *Colletotrichum gossypii* and *Phytophthora parasitica* spores were sprayed on detached leaves. Visually, the detached leaves of the transgenic plants exhibited more resistance to both *C. gossypii* and *P. parasitica* infection than the WT plants (Figure 5A). To quantify the lesions, the number and diameter of the disease spots in the wild-type and transgenic plants were measured, and the data were consistent with what was observed visually (Figure 5B, C).

### Overexpression of *GhWRKY15* affects the expression of PR genes and ethylene (ET) biosynthesis-related genes

**Figure 6:**
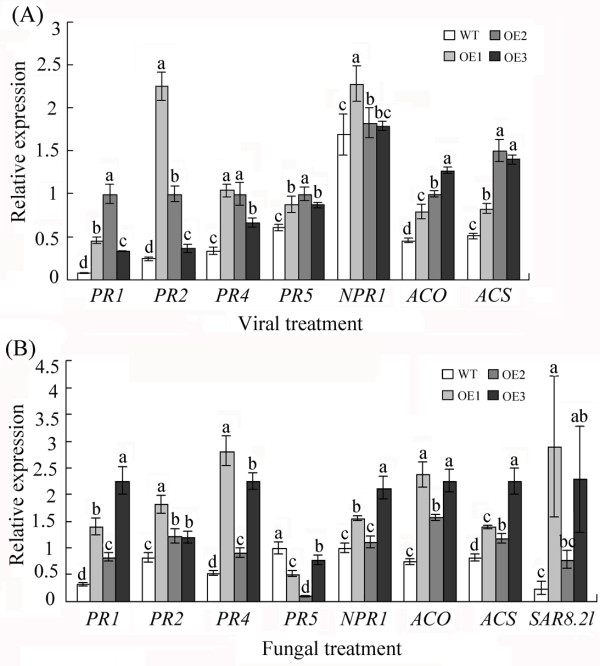
**Expression of defence-related genes and ET biosynthesis genes.** (**A**) The expression of defence-related genes and ET biosynthesis genes was examined following TMV infection. Next, qPCR was used to examine the expression of defence-related genes, including *PR1*, *PR2*, *PR4*, *PR5* and *NPR1*, and ET biosynthesis genes, including *ACO* and *ACS* genes, in plants 10 days post-infection with TMV. (**B**) The expression of defence-related genes and ET biosynthesis genes 7 days post infection with a fungus. The *actin* gene was used to normalise the amount of template in each reaction. The data are presented as the mean ± standard error of three independent experiments. The values indicated by the different letters are significantly different at *P* < 0.05, as determined using Duncan’s multiple range test.

Because many WRKY transcription factors have been shown to be involved in activating the transcription of defence genes, particularly the transcription of PR genes [3], the expression of PR genes was investigated using qPCR after the plants were infected with TMV or *C. gossypii*. Following infection with TMV, the expression of PR genes, including *PR1*, *PR2* (*β-1,3-glucanase*), *PR4* and *PR5* (osmotin), was greater in the three transgenic lines than the expression in the WT plants (Figure 6A). Moreover, the expression of the SA signalling-related gene *NPR1*[3,22] was also slightly enhanced. To further elucidate the possible mechanisms underlying the enhanced viral resistance of the transgenic plants, we examined the expression of two enzymes that are involved in ET biosynthesis, 1-aminocyclopropane-1-carboxylic acid (ACC) synthase (ACS), which catalyses the conversion of *S*-adenosyl-L-Met into ACC, and ACC oxidase (ACO), which converts ACC into ET [23]. In all three transgenic lines, the expression levels of ACO and ACS were higher than those in WT plants infected with TMV (Figure 6A). Similar results were obtained following *C. gossypii* infection, except that the transcription of *PR5* was inhibited in the transgenic plants compared with the WT plants (Figure 6B). In addition, the expression of the disease-responsive gene *SAR8.2 l* was significantly upregulated (Figure 6B). 

### Overexpression of *GhWRKY15* decreases the accumulation of ROS and activates the expression of oxidation-related genes

**Figure 7:**
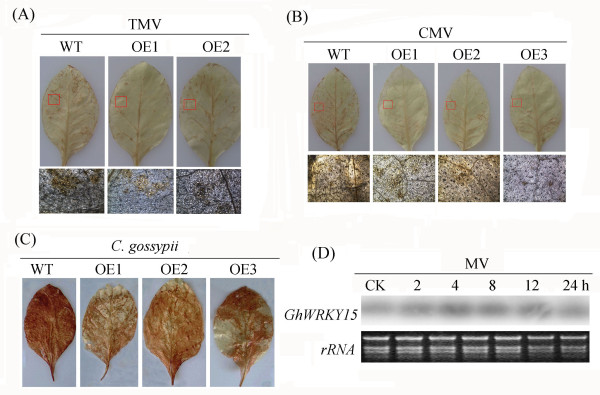
**Expression of *GhWRKY15 *in tobacco decreased the accumulation of ROS, and MV enhanced *GhWRKY15 *expression.** (**A**), (**B**) and (**C**) show that the expression of *GhWRKY15* in tobacco decreased the accumulation of ROS after TMV, CMV or *C. gossypii* treatment, respectively. The level of H_2_O_2_ in the tobacco leaves was determined using 1 mg/ml DAB as substrate. The top figure indicates the visualisation of the H_2_O_2_ accumulation, and the bottom figure shows the microscopic observations of the brown precipitate. (**D**) MV enhances *GhWRKY15* expression. Approximately one-week-old cotton seedlings were used for the 0.5 mM MV treatment. Ethidium bromide-stained rRNA was included as a loading control.

Some virus-host plant interactions result in oxidative stress and the generation of ROS [24]. H_2_O_2_, singlet oxygen, O_2_^-^, and hydroxyl radicals are all ROS, and we monitored the accumulation of H_2_O_2_ in transgenic plants and WT plants following exposure to TMV, CMV or *C. gossypii*. The accumulation of H_2_O_2_ was detected using histochemical analyses via DAB staining. As seen by the visible accumulation of a brown precipitate, the leaves of transgenic tobacco plants accumulated lower levels of H_2_O_2_ relative to the WT plants (Figure 7A, B, C). A microscopic analysis of the plants following viral infection also indicated less brown precipitate on the leaves of transgenic plants when compared with WT plants (Figure 7A, B), which is consistent with the results defined visually. 

**Figure 8:**
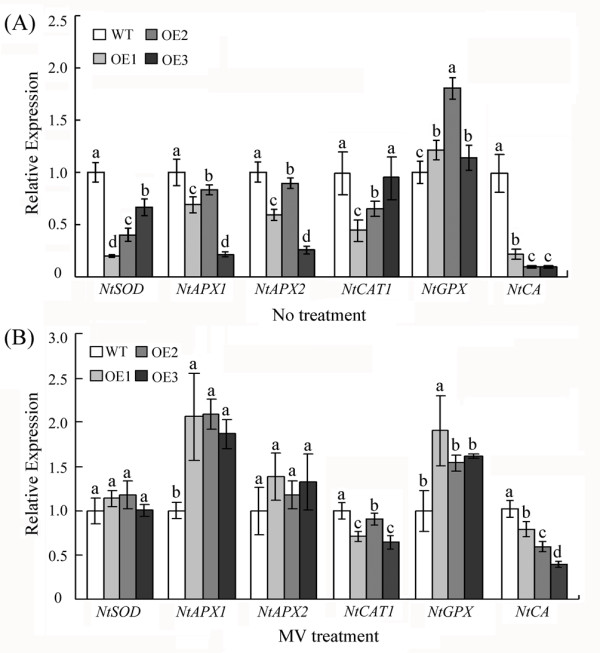
**Expression of antioxidant enzymes in transgenic lines.** (**A**) The expression of antioxidant enzymes under normal conditions. (**B**) The expression of antioxidant enzymes during MV treatment. Also, qPCR analysis was performed to detect the levels of the antioxidant enzymes (*NtSOD*, *NtGPX*, *NtAPX1*, *NtAPX2*, *NtCAT1* and *NtCA*). Approximately three-week-old transgenic and wild-type tobacco plants were used for the expression analysis. For the MV treatment, the tobacco seedlings were sprayed with 0.5 mM MV and analyzed 6 h after treatment. The data are presented as the mean ± standard error of three independent experiments. The values indicated by the different letters are significantly different at *P* < 0.05, as determined using Duncan’s multiple range tests.

**Figure 9:**
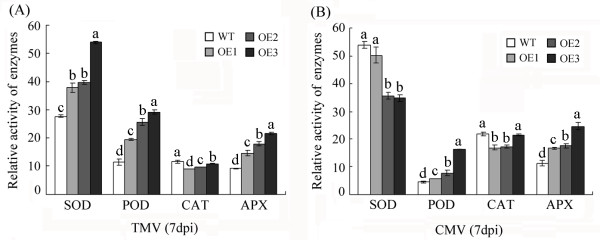
**Effect of virus infection on the SOD, POD, CAT and APX activities.** (**A**) and (**B**) present the SOD, POD, CAT and APX activities 7 days post inoculation with TMV and CMV. The data are presented as the mean ± standard error of three independent experiments. The values indicated by the different letters are significantly different at *P* < 0.05, as determined using Duncan’s multiple range tests.

A potential redox mediator, methyl viologen (MV), was used to verify whether *GhWRKY15* is responsive to oxidative stress. The expression of *GhWRKY15* was upregulated 2 h after treatment with 0.5 mM MV, and it was significantly enhanced at 4 h (Figure 7D and Additional file 5: Figure S4). To explore the underlying mechanism of the reduced ROS levels in the transgenic plants during the disease resistance process, the expression of six genes was analysed in response to oxidative stress. The enzyme SOD acts as the first line of defence against ROS by transforming O^2-^ into H_2_O_2_, and such enzymes as APX, CAT and GPX play important roles in regulating intracellular H_2_O_2_ levels [25,26]. To our surprise, with the exception of the upregulation of *NtGPX*, the expression of the other five antioxidant genes (*NtSOD*, *NtAPX1*, *NtAPX2*, *NtCAT1* and *NtCA*) was decreased in the transgenic plants relative to the WT plants under normal conditions (Figure 8A). However, when treated with MV, the expression of *NtAPX1* and *NtGPX* was enhanced (Figure 8B) in the transgenic plants compared with the wild-type plants, whereas the *NtCAT1* and *NtCA* expression levels were still decreased in the transgenic plants relative to the wild-type plants. We observed no significant differences in the expression of *NtSOD* and *NtAPX2* between the transgenic and WT lines. These results suggest that the overexpression of *GhWRKY15* indeed affected the antioxidant system in tobacco, which led us to further investigate how *GhWRKY15* regulated the ROS scavenging pathway. Thus, we examined the enzymatic activities of SOD, POD, CAT and APX in the oxidative response during treatment with TMV or CMV (Figure 9A, B). Compared to the activities observed in WT plants, the activities of POD and APX were significantly increased in the OE lines, but there was a slight decrease in CAT activity. Interestingly, the activity of SOD was significantly increased following infection with TMV, whereas the activity of SOD following infection with CMV was decreased. These results indicate that the role of GhWRKY15 in the ROS-scavenging pathway is complicated. 

### Expression of *GhWRKY15* affects plant growth and development

**Figure 10:**
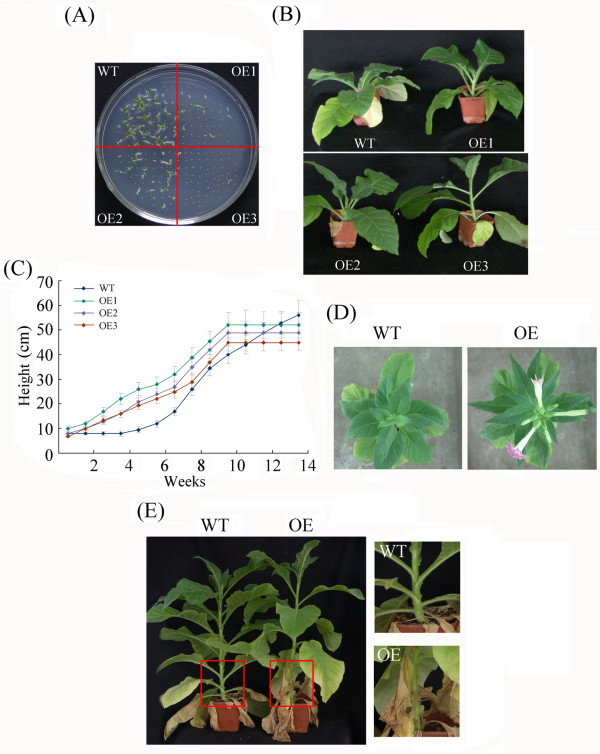
**Comparison of the growth and development of the transgenic and wild-type tobacco.** (**A**) Seed germination and growth phenotype of transgenic and wild-type tobacco. (**B**) The growth phenotype of transgenic and wild-type tobacco at approximately 10 weeks. Differences in stem elongation are clearly observable. (**C**) The height of transgenic and wild-type tobacco from the shooting stage to the flowering stage. (**D**) Premature flowering of the transgenic plants relative to the wild-type plants. The growth phenotype was photographed at approximately 22 weeks. (**E**) The phenotype of the bottom leaves of the transgenic and wild-type tobacco at approximately 18 weeks. The figures are a magnification of the *red boxes* in (E).

**Figure 11:**
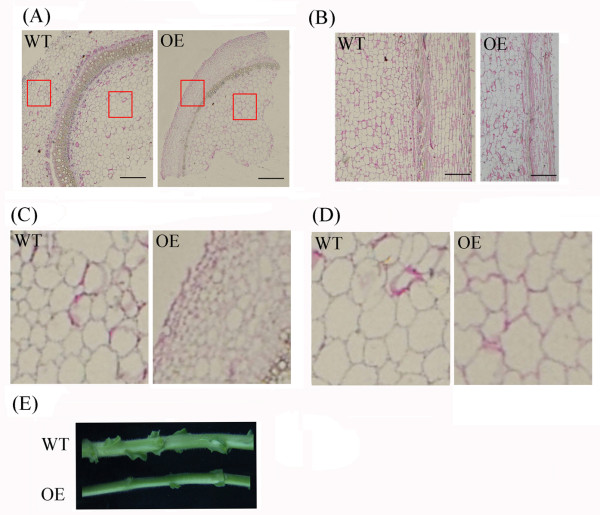
**Comparison of stems between transgenic and wild-type tobacco.** (**A**) Transverse section of the stems of transgenic and wild-type tobacco at the shooting stage. (**B**) Vertical section of the stems of transgenic and wild-type tobacco at the shooting stage. (**C**) Magnification of the *red boxes* on the left in (A). (**D**) Magnification of the *red boxes* on the right in (A). The left and right red boxes primarily indicate cells of the cortex, vascular bundle and pith. Bar: 100 μm. (**E**) Visual differences in the stems of transgenic and wild-type tobacco at the shooting stage.

Many WRKY genes have been reported to affect plant development and growth throughout the entire development and growth period. To investigate the effects of constitutive overexpression of *GhWRKY15* on plant growth and development in tobacco, the growth phenotype of both WT plants and transgenic lines was monitored. Except for the later germination of the transgenic plants relative to the WT plants (Figure 10A), we observed no difference between the plants until approximately ten weeks. The transgenic plants entered the shooting stages earlier than did the wild-type plants. As shown in Figure 10B, the stems of the OE plants began to elongate at approximately ten weeks, but the stems of the WT tobacco had no evident elongation. Interestingly, the stems of the transgenic plants were much thinner than the stems of the WT plants during the shooting stage (Figure 11E). Microscopic analyses revealed that the cortical cells of the transgenic plants had smaller diameters relative to the cells of the WT plants (Figure 11C), but no difference in the cell diameter of the pith was observed (Figure 11D). Moreover, no difference was observed in the length of the cells in the WT and transgenic plants (Figure 11B). After the shooting stage of the transgenic plants began, overexpression of *GhWRKY15* resulted in higher transgenic plants relative to wild-type plants (Figure 10C). However, this difference in the stem length disappeared at the flower stage. Moreover, *GhWRKY15*-overexpressing transgenic plants displayed premature flowering by two to three weeks relative to the wild-type plants under normal growth conditions (Figure 10D). Additionally, the bottom leaves of the OE lines exhibited senescence earlier than the bottom leaves of the WT plants (Figure 10E).

### Tissue-specific expression of *GhWRKY15* and the effects of abiotic stresses on *GhWRKY15* expression

**Figure 12:**
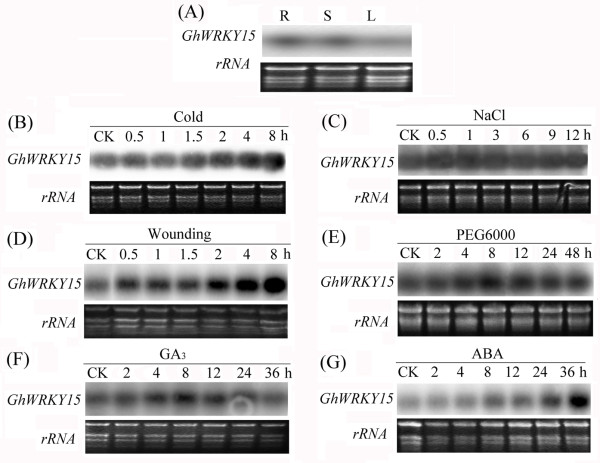
**Tissue-specific expression of *GhWRKY15 *and expression analysis of *GhWRKY15 *in response to abiotic stresses.** Total RNA was extracted from the roots (R), stems (S) and leaves (L) for the tissue-specific expression analysis (**A**). Total RNA was extracted from the leaves at the indicated time points after treatment with cold (4 °C) (**B**), 200 mM NaCl (**C**), wounding (**D**), 15% (w/v) PEG6000 (**E**), 500 μM GA_3_ (**F**) or 100 μM ABA (**G**). Ethidium bromide-stained rRNA was included as a loading control.

To characterise the expression of *GhWRKY15* under abiotic stresses, including cold, high salinity, wounding and drought (PEG6000), northern blots were performed using total RNA extracted from cotton seedlings. Whereas the expression of *GhWRKY15* exhibited negligible changes following NaCl treatment (Figure 12C), the other three treatments resulted in a dramatic increase in the expression of *GhWRKY15* within 8 h. As shown in Figure 12B, D, E, the expression patterns were different. *GhWRKY15* expression increased sharply within 2 h following wounding, and the high level due to the wounding was present until 8 h. Following the cold treatment, increased GhWRKY15 expression was detected at 2 h, and this expression continued gradually to increase. The peak expression of *GhWRKY15* following drought treatment occurred at 8 h, after which the expression level decreased gradually. These results indicate that *GhWRKY15* expression can be regulated by abiotic stresses. Intriguingly, there was a greater accumulation of *GhWRKY15* transcripts in the root and stem than in the leaves (Figure 12A), and GA_3_ treatment led to a higher expression level from 4 h to 24 h (Figure 12F), whereas ABA did not increase expression until 24 h (Figure 12G). These results suggest that *GhWRKY15* may play a role not only in response to abiotic stresses but also in plant growth and development.

## Discussion

Although five major families of plant transcription factors, including bZIP, WRKY, MYB, EREBF and homeodomain protein, have been shown to play roles in the regulation of the plant defence response [2], little is known about the exact function or mechanism of individual transcription factors. Generally, it is believed that the WRKY family of transcription factors plays major roles in plant responses to biotic and abiotic stresses and during development [3,10,11,13,19]. Most functional analyses of WRKY transcription factors have been restricted to *A. thaliana* and *O. sativa*, and few WRKY family members from cotton have been reported previously. To explore the function of the WRKY transcription factors in cotton, we isolated a WRKY gene from *G. hirsutum*, which is an important economic crop cultivated worldwide.

Due to the high similarity between the sequence of the protein encoded by the WRKY gene obtained from cotton and those of several WRKYs obtained from *N. tabacum*, *A. thaliana*, *Solanum tuberosum* and *Populus tomentosa*, we verified that the gene we isolated from cotton is a WRKY transcription factor gene. The phylogenetic tree and the short conserved HARF structural motif further indicated that the *GhWRKY* gene belongs to subgroup IId, with *AtWRKY15*, *AtWRKY11* and *AtWRKY7* as its closest homologues in *A. thaliana*[20]; As a result, we designated the isolated gene *GhWRKY15*. Consistent with the putative role of WRKY proteins as transcription factors, a nuclear targeting sequence (KKRK) was identified in the C-terminal region of the protein, and the targeting experiment suggested that GFP-tagged GhWRKY15 was located in nucleus. These results suggest that, similar to *GhWRKY3*[27], *GhWRKY15* might function in the nucleus.

To investigate the effects of constitutive overexpression of *GhWRKY15* on the response to pathogen resistance, *GhWRKY15* overexpressing-tobacco plants were inoculated with two types of viruses and two pathogenic fungi. The plants constitutively overexpressing *GhWRKY15* were more resistant to TMV and CMV infection compared with the WT tobacco plants. This observation diverges from previous research demonstrating that the virus-induced gene silencing (VIGS) of *NbWRKY1*, *NbWRKY2*, or *NbWRKY3* suppressed resistance to TMV and that the size of the hypersensitive response (HR) lesions was also reduced in *NbWRKY1*-silenced plants [22]. The *GhWRKY15* transgenic plants also exhibited increased resistance to fungal pathogens (*C. gossypii* and *P. parasitica*) compared with wild-type plants. Previously, overexpression studies examining a pair of allelic genes *OsWRKY45-1* and *OsWRKY45-2* demonstrated that these genes positively regulated resistance to the rice fungal pathogen *Magnaporthe grisea*[28]. Our data presented above indicate that *GhWRKY15* overexpression increases the tolerance of the plant to pathogens; therefore, the upregulation of *GhWRKY15* in cotton seedlings in response to pathogens, including *R. solani*, *C. gossypii* and *F. oxysporum*, suggests that GhWRKY15 may participate in the regulation of plant responses to disease defence. The increased expression of *PR1*, *PR2* and *PR4* contributed to the increased resistance of transgenic *GhWRKY15*-overexpressing plants to TMV and *C. gossypii*. Furthermore, previous studies have demonstrated that WRKY DNA binding-proteins can regulate the transcription of a number of defence-related genes, including the well-studied PR genes, via binding to the W-box in the promoter of these genes [11,29]. It is noted that the expression of the *PR5* gene in transgenic plants during *C. gossypii* infection was down-regulated compared with the infected WT plants, suggesting that the molecular mechanism underlying the activity of *GhWRKY15* is fairly complex under different biotic stresses or that *GhWRKY15* may be an integrator of several signalling pathways. SA and JA are two well-studied key mediators of plant resistance. In *Arabidopsis*, more than 49 WRKY genes can be differentially regulated after infection with *P. syringae* or treatment with SA [30]. Infection of *GhWRKY15* transgenic plants with TMV and *C. gossypii* increased the expression of *NPR1*, which is a key regulator of the SA-dependent defence pathway and SAR. Furthermore, the analysis of the expression of *GhWRKY15* in cotton seedlings showed that *GhWRKY15* expression can be affected by SA and MeJA, supporting our hypothesis that *GhWRKY15* plays a role in resistance to viral and fungal pathogens via SA- and JA-dependent defence pathways. Interestingly, reports of WRKY proteins that function in both SA- and JA-dependent pathways are relatively limited [7]. Moreover, the expression of two key enzymes that are involved in ET biosynthesis (ACO and ACS) is increased in *GhWRKY15* transgenic plants following infection with fungi and viruses. And there was significant increase of *GhWRKY15* expression in cotton seedlings following ET treatment. This suggests that the increased disease resistance of *GhWRKY15* overexpressing tobacco compared to the wide type may partially depend on the expression of ET synthesis related genes. Additionally, *GhWRKY15* expression could be increased under various abiotic stress conditions, including low temperature, wounding and drought, and a group of *cis*-elements related to biotic and abiotic stresses were identified in the *GhWRKY15* promoter. Altogether, these data support our hypothesis that the mechanism of response to various environmental stresses, especially viral and fungal infection, in *GhWRKY15* overexpressing plants may be related with multiple signalling pathways, including SA, JA or ET signalling pathways.

Mounting evidence suggests that the ROS network is essential for the induction of disease resistance [31]. For instance, mitogen-activated protein kinase (MAPK) cascades members *GhMAPK2* and *GhMAPK6* are both involved in the response to infection and oxidative stress [32,33]. Following challenge with a virus or a fungus, a lower accumulation of H_2_O_2_ and a higher activity of antioxidant enzymes, including POD and APX, were detected in the *GhWRKY15*-overexpressing plants relative to the WT plants. We reasoned that *GhWRKY15* upregulates the activity of POD and APX to increase the oxidative stress tolerance and contributes to the resistance towards biotic stresses in transgenic plants. However, except for *NtGPX*, the expression of the other five antioxidant enzymes examined was reduced in unstressed transgenic seedlings compared with the wild-type plants. These results contrast previous research examining some genes, which appear to play positive roles in the stress response and enhance the expression of most antioxidant enzymes [32,34]. To explain this difference, the expression of the six antioxidant enzymes was examined during treatment with MV, which is a potential redox mediator. Although there was no significant difference in the expression of *NtSOD* and *NtAPX2* in the transgenic plants compared with the WT plants following the treatment with MV, the expression of these genes was increased compared with the expression in plants receiving no treatment. Additionally, the upregulation of *NtGPX* and *NtAPX1* expression during MV treatment suggests that *GhWRKY15* overexpression can help plants maintain ROS homoeostasis under oxidative stresses to alleviate injury to the organism. Most recently, it has been reported that there was a substantial increase in the *MusaWRKY71* level when banana plantlets were subjected to H_2_O_2_. Moreover, a putative thioredoxin-dependent peroxidase gene belonging to the ROS detoxification system was induced in *MusaWRKY71*-overexpressing cultures [35]. Furthermore, in *Arabidopsis*, the maximum expression of *WRKY53* was detected in seven-week-old plants overexpressing *WRKY53*, at which point the H_2_O_2_ level increased significantly [18]. In wild-type cotton seedlings, a substantial increase in the level of *GhWRKY15* was also observed following exposure to MV.

Intriguingly, some of the growth and development phenomena observed in plants overexpressing *GhWRKY15* are similar to those observed in *AtWRKY53* over-expressing plants. Similar to *GhWRKY15-*overexpressing plants, *AtWRKY53*-overexpressing plants demonstrated early flowering after sowing, and the senescence of the whole plant with partly necrotic leaves was visible earlier when compared with wild-type plants [18]. Interestingly, overexpression of *GhWRKY15* in *N. tabacum* cv. NC89 promoted stem elongation and inhibited the transversal growth of stems. *AtWRKY12* controls cell fate in thickened pith cells by acting as a negative regulator of NST2 and C3H zinc finger TFs, which, in turn, regulate secondary cell wall synthesis [19]. Although no difference in the diameter and length of pith cells was observed in our transgenic plants, overexpression of *GhWRKY15* may affect the fate of cells in the cortex, which had a smaller diameter relative to WT plants. The phytohormone GA is an essential regulator of stem elongation and other developmental processes throughout the life cycle of a plant [36]. The induction of *GhWRKY15* expression during GA_3_ treatment combined with the higher expression of *GhWRKY15* in the roots and stems compared with the cotyledon of cotton may clarify the underlying mechanism of the influence of *GhWRKY15* overexpression on plant growth and development.

## Conclusion

In conclusion, we identified and characterised a group IId WRKY member in cotton. Our results strongly suggest that overexpression of *GhWRKY15* confers tobacco resistance to both viral and fungal pathogens, and indicate that overexpression of *GhWRKY15* regulates the stem growth or development in tobacco. The elucidation of the regulatory mechanism of overexpression of *GhWRKY15* in tobacco may provide a converging node of regulatory pathways in response to pathogens and oxidative stress and in the regulation of plant development. Although the influence of overexpression of *GhWRKY15* in tobacco was explored, the mechanism underlying the function of GhWRKY15 should be elucidated in transgenic cotton and loss-of function data in cotton are particularly needed. Moreover, other research focusing GhWRKY15 is still needed, for example, the study of its interactions with other WRKY proteins or MAPK cascades members.

## Methods

### Plant growth and treatments

Cotton (*Gossypium hirsutum* L. cv Lumian 22) seedlings were grown in a growth chamber at 28 °C under a 16 h light and 8 h dark photoperiod with a light intensity of 300 μmol m^-2^ s^-1^. Seven-day-old cotton seedlings were collected for the various treatments. For the tissue-specific expression analysis, the roots, stems and leaves were harvested separately from seven-day-old cotton seedlings. For the hormone treatments, the leaves of uniformly developed seedlings were sprayed with 2 mM SA, 100 μM ABA, 100 μM methyl jasmonate (MeJA), ET released from 5 mM ethephon, 500 μM GA_3_, or 0.5 mM methyl viologen (MV). For the salt and drought treatments, the seedlings were cultured in solutions containing 200 mM NaCl or 15% (w/v) PEG6000, respectively. The mechanical wounding procedure was performed on leaves of seedlings by crushing the leaves with a hemostat, and a low temperature of 4 °C was used for the cold treatment. For the fungal pathogen treatment, the roots of cotton seedlings were dipped into *Fusarium oxysporum* f. sp. *vasinfectum*, *Rhizoctonia solani*, or *C. gossypii* conidial suspensions (10^5^ conidia/ml). The cotyledons, leaves, stems and roots were harvested, frozen directly in liquid nitrogen and stored at −80 °C for later use.

### Cloning of the full-length *GhWRKY15* cDNA

Total RNA was extracted from the leaves of seven-day-old cotton seedlings 8 h after treatment with 2 mM SA treatment using Trizol Reagent (Invitrogen, USA). Reverse transcription-PCR (RT-PCR) and RACE-PCR were used to amplify the full-length *GhWRKY15* cDNA. The general PCR procedures and primers (WQ1 and WQ2) are shown in Additional file 1 (Table S1 and Table S2), respectively. The PCR product was purified, cloned into the pMD18-T vector, and transformed into competent *Escherichia coli* cells for sequencing. The amino acid sequence alignment of *GhWRKY15* and its homologues was conducted using BLAST ( http://www.ncbi.nlm.gov/blast) and DNAman software 5.2.2 (Lynnon Biosoft, Canada). A phylogenic analysis of the GhWRKY15 protein and other WRKYs was performed in MEGA using the neighbour-joining method.

### Amplification of the *GhWRKY15* genomic sequence and promoter

Genomic DNA was isolated from seedling leaves using the revised CTAB method. One pair of primers (WG1 and WG2), which was designed and synthesised based on the full-length *GhWRKY15* cDNA, was used to amplify the *GhWRKY15* genomic sequence using genomic DNA as the template. I-PCR was performed to obtain the promoter sequence. Three restriction endonucleases (*Dra*I, *Taq*I and *Vsp*I) were used to digest the cotton seedling genomic DNA, and T4 DNA ligase (TaKaRa, China) was used to self-ligate the DNA fragments into circles, which were used as templates to amplify the promoter region. Three promoter fragments were amplified using six pairs of primers. Using nested PCR, the first fragment was obtained using two pairs of primers, Dra1/Dra2 and Dra3/Dra4, and a *Dra*I-digested template. Based on the obtained sequences, the primer pairs Taq1/Taq2, Taq3/Taq4, Vsp1/Vsp2, and Vsp3/Vsp4 were used to sequentially amplify the other two fragments using the same method. The deduced portion of the promoter with the three fragments was subsequently verified using the special primers WP1 and WP2. The sequences of the primers are provided in Additional file 1 (Table S2). The programme PlantCARE ( http://bioinformatics.psb.ugent.be/webtools/plantcare/html) was used to analyse the *GhWRKY15* promoter sequence.

### Subcellular localisation of GhWRKY15

To construct the pBI121-GhWRKY15-GFP expression plasmid driven by the cauliflower mosaic virus (CaMV) 35S promoter, the coding sequence of *GhWRKY15* without a termination codon was amplified using PCR and the primers Wsb-1 (5'-GGATCCCATCTTTTCCTAATGTGGGAT-3', *Bam*HI site underlined) and Wsb-2 (5'-CTCGAGTGAAGATTCAAGTATGGTGG-3', *Xho*I site underlined). This fragment was subsequently fused to the N-terminus of GFP (green fluorescent protein). The resulting expression plasmid, pBI121-GhWRKY15-GFP, or the pBI121-GFP control plasmid was transformed into onion (*Allium cepa*) epidermis cells via biolistic bombardment transformation using the Biolistic PDS-1000/He system (Bio-Rad, USA) with gold particles (1.0 μl) and a helium pressure of 1,350 psi. After incubation of the transformed onion epidermis cells at 25 °C for 8 h in the dark, the nuclei were stained with 100 μg/ml of 4',6-diamidino-2-phenylindole (DAPI) (Solarbio, China) in phosphate-buffered saline for 10 min, and the onion epidermal cells were visualised using a fluorescence microscope (BX51, model 7.3; Japan).

### Northern blot analysis

Total RNA was extracted using an RNeasy Mini Kit (Qiagen, USA), as recommended by the manufacturer, fractionated in a 1.0% agarose gel containing formaldehyde and blotted onto a Hybond-N nylon membrane. A fragment amplified using the two specific primers WN1 and WN2, which were designed based on the *GhWRKY15* cDNA sequence, was used as a probe, and northern blot hybridisations were performed as described by [37].

### Vector construction and transgenic plant generation

The full-length coding sequence of *GhWRKY15* amplified using the primers WE1 (5'-GGATCCCATCTTTTCCTAATGTGGGAT-3') with a *Bam*HI site and WE2 (5'-GAGCTCCTATGAAGATTCAAGTATGGTGG-3') with a *Sac*I site was inserted into the same sites behind the CaMV35S promoter in the pBI121 binary vector. The ligated construct (pBI121-*GhWRKY15*) was electroporated into *Agrobacterium tumefaciens* (strain LBA4404) and transformed into tobacco (*Nicotiana tabacum* cv. NC89) using the leaf disk method as described previously [38]. The T_0_ and T_1_ transgenic tobacco plants were verified by PCR with special primers, which were designed according to the full-length coding sequence of *GhWRKY15*. The transgenic T_2_ lines were used in the experiments.

### Disease resistance of transgenic tobacco plants

*C. gossypii* and *P. parasitica* spore suspensions (10^6^ conidia/ml) prepared in 1% glucose were sprayed onto the leaves of seven-week-old *GhWRKY15* transgenic tobacco plants and wild-type plants. The inoculated plants were maintained in the growth chamber at 25°C for 24 h in the dark and were incubated at 25 °C under a 16 h light/8 h dark period. The inoculations with TMV and CMV were performed using a standard mechanical rubbing method, as described in [33]. The inoculated plants were cultured in the greenhouse at 25 °C under a 16 h light/8 h dark period.

### Virus quantitation

Polyclonal antisera specific for the CP of TMV and CMV was used to detect virus accumulation in the tobacco leaves. The proteins in the inoculated leaves were extracted 10 days post-infection. Alkaline phosphatase-conjugated goat anti-rabbit immunoglobulin G (Promega, USA) was used as the secondary antibody, and the plates were developed using a *p*-nitrophenyl phosphate (Sigma, USA) solution as a substrate. The absorbance of the developed plates was read at 490 nm using a Bio-Rad ELISA reader.

### Histochemical detection of H_2_O_2_ and enzyme activity assays

Using 3,3-diaminobenzidine (DAB) as a substrate, H_2_O_2_ was visually detected in the leaves of the plants [39]. After various treatments, the leaves of the transgenic and wild-type tobacco plants were immersed in a 1 mg/ml DAB solution for 6 h in the dark and subsequently treated with 95% ethanol to remove the chlorophyll. This treatment decolourises the leaves, except for the deep-brown stain produced by the polymerisation of DAB following interaction with H_2_O_2_. The leaves of the transgenic and WT plants inoculated with TMV or *C. nicotianae* were collected to measure the SOD, POD, CAT and APX activity, as described previously [40].

### Fluorescence real-time qPCR

Fluorescence real-time qPCR was performed using cDNA as the template, which was synthesised from total RNA extracted from the transgenic or WT tobacco lines. The amplified fragments were all shorter than 300 bp. The qPCR technique was performed using the SYBR PrimeScript RT-PCR Kit (TaKaRa, China) and a CFX96TM Real-Time System (Bio-Rad, USA), as recommended by the manufacturer. The PCR programme was as follows: pre-denaturation at 95°C for 30 s; 40 cycles of 95°C for 30 s, 53°C for 15 s and 72°C for 15 s; and a melt cycle from 65°C to 95°C. The expression of *actin* was used as an internal control. The significance of the differences was determined using Statistical Analysis System (SAS) version 9.1 (SAS Institute, USA). To determine the copy number, qPCR was also used, as described by [41].

### Expression analysis of defence-related genes in transgenic and WT lines

The qPCR technique was used to analyse the expression of PR genes, ET biosynthesis-related genes and oxidation-related genes. The GenBank accession numbers of the defence-related genes examined in the qPCR analysis are as follows: X63603 (*actin*), X06361 (*NtPR1*), M60460 (*NtPR2*), X58546 (*NtPR4*), M29279 (*NtPR5*), DQ837218 (*NtNPR1*), NTU96152 (*SAR8.2°l*), AB012857 (*NtACO*), AJ005002 (*NtACS*) AB093097, (*NtSOD*), AU15933 (*NtAPX1*), D85912 (*NtAPX2*), U93244 (*NtCAT1*), AB041518 (*NtGPX)* and AF454759 (*NtCA*).

## Authors’ contributions

FY carried out most of the experiments, and drafted the manuscript. YH participated in RNA blot analysis and function analysis of the transgenic plants. WL helped to revise the manuscript. CW, XC and XG conceived the experimental design and helped to draft the manuscript. All authors read and approved the final manuscript.

## Supplementary Material

Additional file 1 **Table S1.** Polymerase chain reaction amplification conditions. **Table S2** Primers used in this study. **Table S3** Ct and Tm value of *GhRDR6* and *GhWRKY15* genes in cotton. **Table S4** Estimation of copy number of *GhWRKY15* gene in cotton. Click here for file

Additional file 2 **Figure S1.** Schematic representation of the locus. The lengths of the exons and introns of *GhWRKY15* (GenBank accession number: GU207869), *AtWRKY7* (NC_003075), *AtWRKY15* (NC_003071) and *VvWRKY* (NW_002239918) are indicated according to the scale below. Exons and introns are designated using *white* or *gray bars*, respectively. The untranslated regions are indicated by black bars. The translation initiation and stop codons are marked with (▽) and (▼), respectively. The R-type introns are indicated by (*). Click here for file

Additional file 3 **Figure S2.** Standard curves of *GhRDR6* and *GhWRKY15*. (A) Standard curves of *GhRDR6* gene from the amplification of six five-fold serial dilutions of plasmid fused by *GhRDR6*. (B) Standard curves of *GhWRKY15* gene from the amplification of six five-fold serial dilutions of the same plasmid fused by *GhWRKY15*. Correlation coefficient and slope values are indicated. The calculated threshold cycle values were plotted versus the log of each starting quantity. Click here for file

Additional file 4 **Figure S3.** Relative expression of *GhWRKY15* in response to different fungal infections and hormone treatments. The results correspond to the results in Figure 3. Transcriptional levels of *GhWRKY15* under different fungal infections and hormone treatments are indicated relative to the level of wild-type cottons without any treatment taken as 1 in each experiment. Click here for file

Additional file 5 **Figure S4.** MV enhances *GhWRKY15* expression. The result corresponds to the results in Figure 7D. Transcriptional levels of *GhWRKY15* under 0.5 mM MV treatment are indicated relative to the level of wild-type cottons without any treatment taken as 1. Click here for file
