# Supplementary material for: GhWRKY15, a member of the WRKY transcription factor family identified from cotton (Gossypium hirsutum L.), is involved in disease resistance and plant development
Source: BMC Plant Biol. 2012 Aug 12;12:144. doi: 10.1186/1471-2229-12-144 (PMC3489871; doi:10.1186/1471-2229-12-144)
Supplement: Additional file 1 — Table S1. Polymerase chain reaction amplification conditions. Table S2 Primers used in this study. Table S3 Ct and Tm value of GhRDR6 and GhWRKY15 genes in cotton. Table S4 Estimation of copy number of GhWRKY15 gene in cotton. [file 1471-2229-12-144-S1.doc]

**Additional files**

**Figure S1** Schematic representation of the locus.

The lengths of the exons and introns of *GhWRKY15* (GenBank accession number: GU207869), *AtWRKY7* (NC_003075), *AtWRKY15* (NC_003071) and *VvWRKY* (NW_002239918) are indicated according to the scale below. Exons and introns are designated using *white* or *gray bars*, respectively. The untranslated regions are indicated by black bars. The translation initiation and stop codons are marked with (▽) and (▼), respectively. The R-type introns are indicated by (*).

**Figure S2 Standard curves of *GhRDR6* and *GhWRKY15*.**

(A) Standard curves of *GhRDR6* gene from the amplification of six five-fold serial dilutions of plasmid fused by *GhRDR6*. (B) Standard curves of *GhWRKY15* gene from the amplification of six five-fold serial dilutions of the same plasmid fused by *GhWRKY15*. Correlation coefficient and slope values are indicated. The calculated threshold cycle values were plotted versus the log of each starting quantity.

**Figure S3** **Relative expression of *GhWRKY15* in response to different fungal infections and hormone treatments**. The results correspond to the results in Figure 3. Transcriptional levels of *GhWRKY15* under different fungal infections and hormone treatments are indicated relative to the level of wild-type cottons without any treatment taken as 1 in each experiment.

**Figure S4 MV enhances *GhWRKY15* expression.** The result corresponds to the results in Figure 7D. Transcriptional levels of *GhWRKY15* under 0.5 mM MV treatment are indicated relative to the level of wild-type cottons without any treatment taken as 1.

**Supplementary Tables:**

**Table S1** Polymerase chain reaction amplification conditions.

| Primers pair | PCR amplification conditions |
| --- | --- |
| DP1/DP2 | 94 °C for 5 min, 35 cycles of 94 °C for 40 s, 50 °C for 40 s and 72 °C for 1 min, then 72 °C for 10 min |
| 5W1/AAP | 94 °C for 5 min, 32 cycles of 94 °C for 40 s, 53 °C for 40 s and 72 °C for 1 min, then 72 °C for 10 min |
| 5W2/AUAP | 94 °C for 5 min, 35 cycles of 94 °C for 40 s, 53 °C for 40 s and 72 °C for 50 s, then 72 °C for 10 min |
| 3W1/B26 | 94 °C for 5 min, 32 cycles of 94 °C for 40 s, 53 °C for 40 s and 72 °C for 1 min, then 72 °C for 10 min |
| 3W2/B25 | 94 °C for 5 min, 35 cycles of 94 °C for 40 s, 53 °C for 40 s and 72 °C for 50 s, then 72 °C for 10 min |
| WQ1/WQ2 | 94 °C for 5 min, 35 cycles of 94 °C for 40 s, 55 °C for 40 s and 72 °C for 1 min, then 72 °C for 10 min |
| WG1/WG2 | 94 °C for 10 min, 35 cycles of 94 °C for 40 s, 54 °C for 40 s and 72 °C for 1 min 30 s, then 72 °C for 10 min |
| Dra1/Dra2  Dra3/Dra4 | 94 °C for 10 min, 32 cycles of 94 °C for 40 s, 53 °C for 40 s and 72 °C for 1 min 30 s, then 72 °C for 5 min  94 °C for 10 min, 35 cycles of 94 °C for 40 s, 55 °C for 40 s and 72 °C for 1 min, then 72 °C for 10 min |
| Taq1/Taq2  Taq3/Taq4 | 94 °C for 10 min, 32 cycles of 94 °C for 40 s, 53 °C for 40 s and 72 °C for 1 min 30 s, then 72 °C for 5 min  94 °C for 10 min, 35 cycles of 94 °C for 40 s, 53 °C for 40 s and 72 °C for1 min, then 72 °C for 10 min |
| Vsp1/Vsp2 | 94 °C for 10 min, 32 cycles of 94 °C for 40 s, 50 °C for 40 s and 72 °C for 1 min 30 s, then 72 °C for 5 min |
| Vsp3/Vsp4 | 94 °C for 10 min, 35 cycles of 94 °C for 40 s, 52 °C for 40 s and 72 °C for1 min, then 72 °C for 10 min |
| WP1/WP2 | 94 °C for 10 min, 35 cycles of 94 °C for 40 s, 52 °C for 40 s and 72 °C for1 min, then 72 °C for 10 min |

**Table S2** Primers used in this study.

| Abbreviation | Primer sequence (5′-3′) | Description |
| --- | --- | --- |
| DP1 | GNACNGGNCAYGCNMGNTTYMG | cDNA sequence primer, forward |
| DP2 | TTYTGNCCRTAYTTNCKCCA | cDNA sequence primer, reverse |
| 5W1 | GAAACGCTGATGAAGATGGTTG | 5′ RACE reverse primer, outer |
| 5W2 | CAGTGGGATCTGTTGAATCGGA | 5′ RACE reverse primer, inner |
| AAP | GGCCACGCGTCGACTAGTAC(G)_14_ | Abridged anchor primer |
| AUAP | GGCCACGCGTCGACTAGTAC | Abridged universal amplification primer |
| 3W1 | CAACCATCTTCATCAGCGTTTC | 3′ RACE forward primer, outer |
| 3W2 | CCTTTCCTCCGCTGGTAAAC | 3′ RACE forward primer, inner |
| B26 | GACTCTAGACGACATCGA(T)_18_ | 3′ RACE universal adaptor primer |
| B25 | GACTCTAGACGACATCGA | 3′ RACE universal primer |
| WQ1 | CATCTTTTCCTAATGTGGGAT | Full-length cDNA primer, forward |
| WQ2 | GATTCAAGTATGGTGGTTTCTGC | Full-length cDNA primer, reverse |
| WG1 | catcttttcctaatgtgggat | Genomic sequence primer, forward |
| WG2 | CAAGGAAATAAACGAGCAGA | Genomic sequence primer, reverse |
| Dra1 | CTGCCGGTGACCACTTATTTAGA | Inverse PCR forward primer, outer |
| Dra2 | CCTTTCCTCCGCTGGTAAACC | Inverse PCR reverse primer, outer |
| Dra3 | CTTGAACTTAGAAACGGCGGCTT | Inverse PCR forward primer, inner |
| Dra4 | TTCGAGACCAGAAGCGGCT | Inverse PCR forward primer, inner |
| Taq1 | GGGAGGGGGTTTGGTTTGTT | Inverse PCR forward primer, outer |
| Taq2 | CAACAGATGCTTAGTTACAGA | Inverse PCR reverse primer, outer |
| Taq3 | GAACCTCGTTAAATCTCTGGT | Inverse PCR forward primer, inner |
| Taq4 | GAAACCGCCGTTCAAGAAGC | Inverse PCR reverse primer, inner |
| Vsp1 | GCTTCTACTTAATAGCCACC | Inverse PCR forward primer, outer |
| Vsp2 | CAACAACTTCCACTTCGTAC | Inverse PCR reverse primer, outer |
| Vsp3 | GCAAACACAGAGCATACTAC | Inverse PCR forward primer, inner |
| Vsp4 | GAGGATTGGAACTGCAAATAC | Inverse PCR reverse primer, inner |
| WP1 | GCCAAGTCTTCGTCTTCAAAAG | Primer of the promoter |
| WP2 | AAAGGGAGGGGGTTTGGTTTG | Primer of the promoter |

**Table S3** Ct and Tm value of *GhRDR6* and *GhWRKY15* genes in cotton

| *Gossypium hirsutum* L. | *GhRDR6* | | *GhWRKY15* | |
| --- | --- | --- | --- | --- |
|  | Cycle threshold (Ct) | Tm (°C) | Cycle threshold (Ct) | Tm (°C) |
| 1 | 23.85 | 82.5 | 23.03 | 83 |
| 2 | 24.95 | 82.5 | 24.23 | 83 |

**Table S4** Estimation of copy number of *GhWRKY15* gene in cotton

| *Gossypium hirsutum* L. | Calculation result of *GhRDR6* gene | Calculation result of *GhWRKY15* gene | *GhWRKY15*/ *GhRDR6* | Copy  number |
| --- | --- | --- | --- | --- |
| 1 | -3.95 | -4.3 | 1.09 | 1 |
| 2 | -4.29 | -4.75 | 1.11 | 1 |
