# Supplementary figures and images for: GhWRKY15, a member of the WRKY transcription factor family identified from cotton (Gossypium hirsutum L.), is involved in disease resistance and plant development
Source: BMC Plant Biol. 2012 Aug 12;12:144. doi: 10.1186/1471-2229-12-144 (PMC3489871; doi:10.1186/1471-2229-12-144)

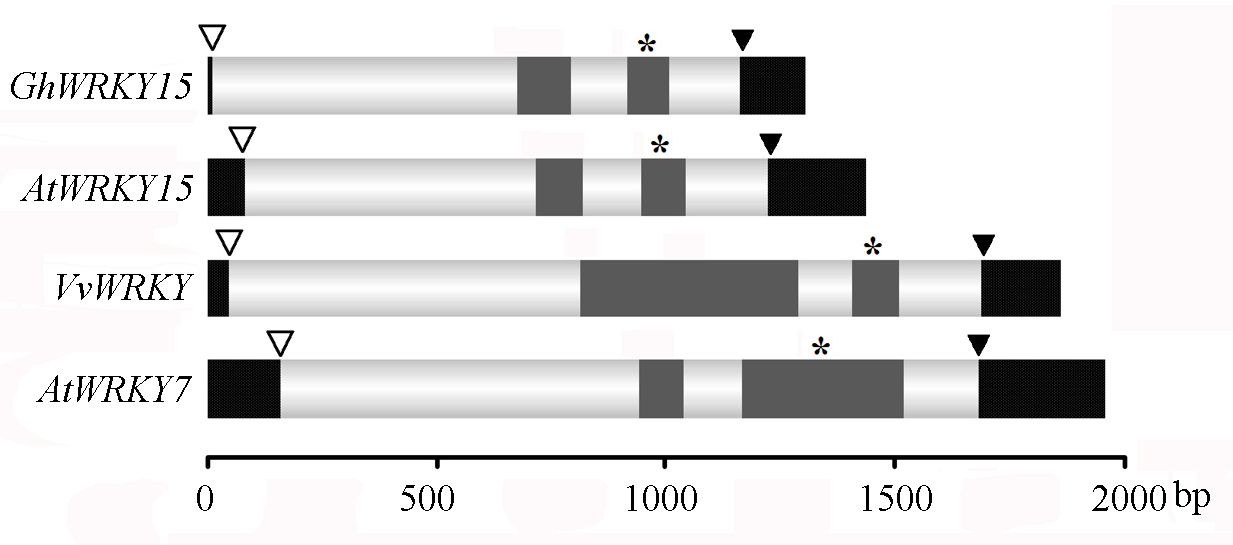

Supplement: Additional file 2 — Figure S1. Schematic representation of the locus. The lengths of the exons and introns of GhWRKY15 (GenBank accession number: GU207869), AtWRKY7 (NC_003075), AtWRKY15 (NC_003071) and VvWRKY (NW_002239918) are indicated according to the scale below. Exons and introns are designated using white or gray bars, respectively. The untranslated regions are indicated by black bars. The translation initiation and stop codons are marked with (▽) and (▼), respectively. The R-type introns are indicated by (*). [file 1471-2229-12-144-S2.tiff]

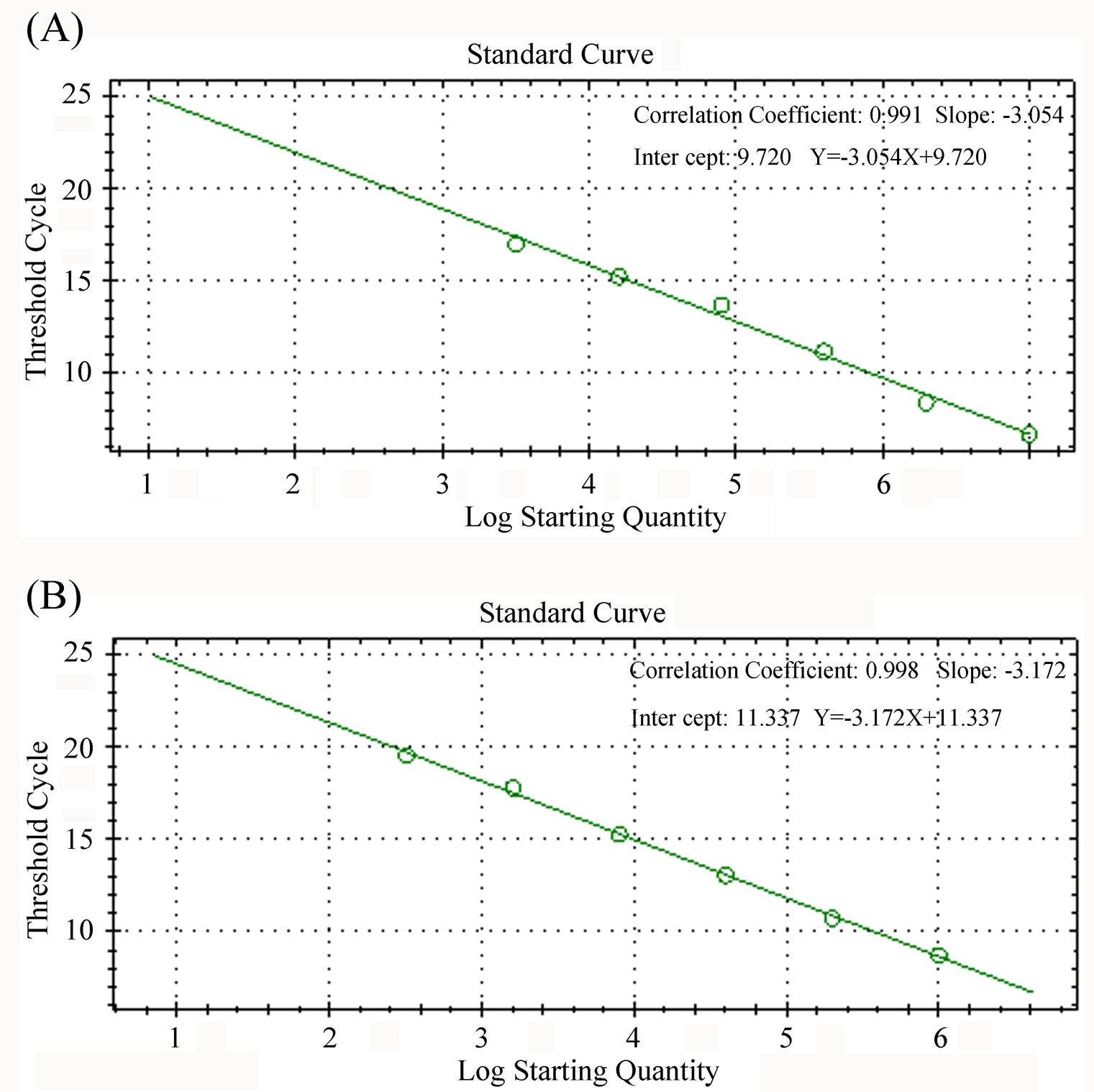

Supplement: Additional file 3 — Figure S2. Standard curves of GhRDR6 and GhWRKY15. (A) Standard curves of GhRDR6 gene from the amplification of six five-fold serial dilutions of plasmid fused by GhRDR6. (B) Standard curves of GhWRKY15 gene from the amplification of six five-fold serial dilutions of the same plasmid fused by GhWRKY15. Correlation coefficient and slope values are indicated. The calculated threshold cycle values were plotted versus the log of each starting quantity. [file 1471-2229-12-144-S3.tiff]

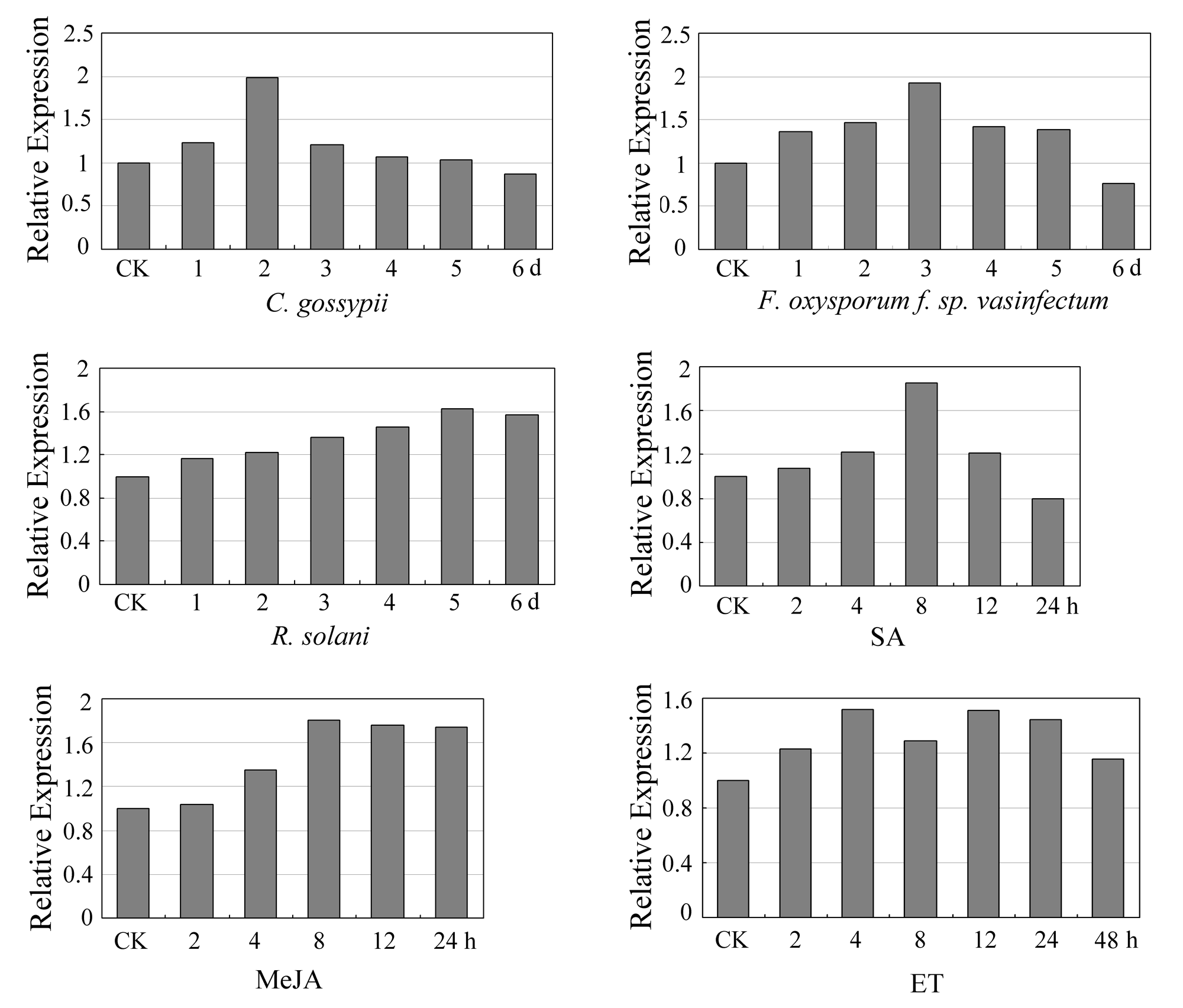

Supplement: Additional file 4 — Figure S3. Relative expression of GhWRKY15 in response to different fungal infections and hormone treatments. The results correspond to the results in Figure 3. Transcriptional levels of GhWRKY15 under different fungal infections and hormone treatments are indicated relative to the level of wild-type cottons without any treatment taken as 1 in each experiment. [file 1471-2229-12-144-S4.tiff]

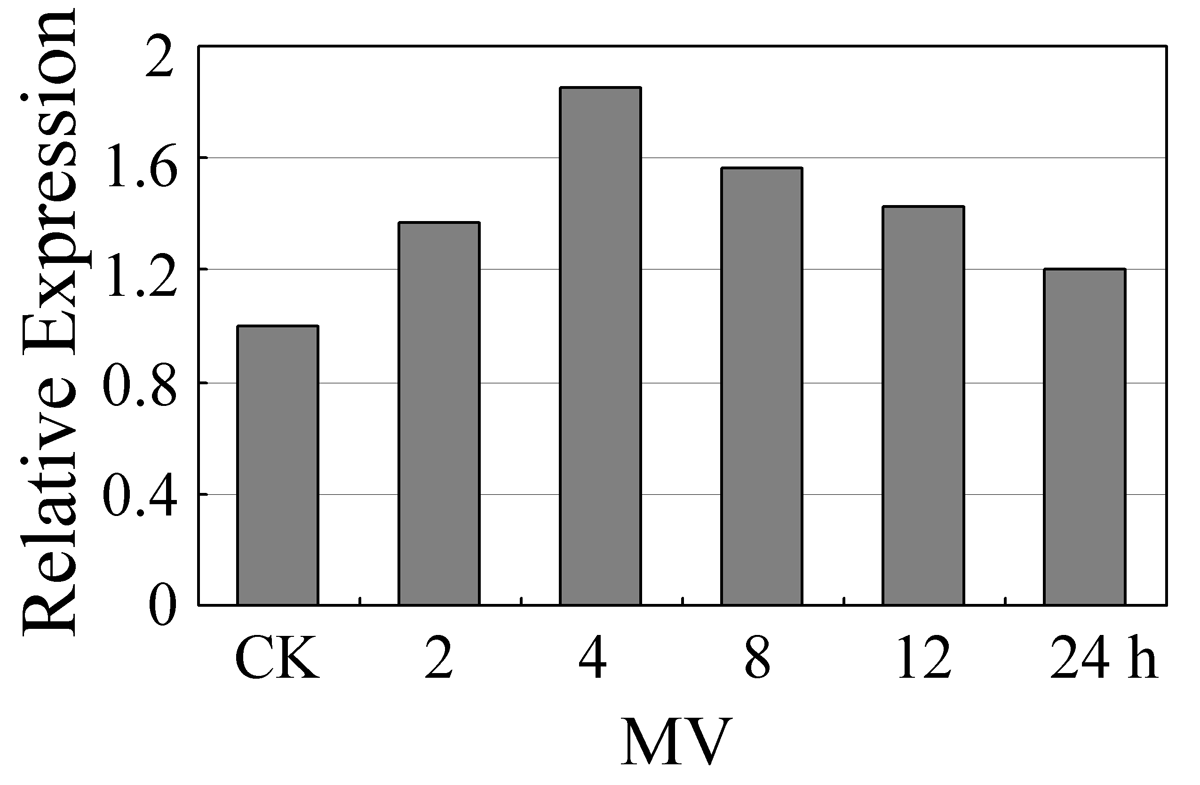

Supplement: Additional file 5 — Figure S4. MV enhances GhWRKY15 expression. The result corresponds to the results in Figure 7D. Transcriptional levels of GhWRKY15 under 0.5 mM MV treatment are indicated relative to the level of wild-type cottons without any treatment taken as 1. [file 1471-2229-12-144-S5.tiff]
